# 1583. A Case Series of Low-Level HBV Viremia After Switching to Long-Acting Injectable Cabotegravir/Rilpivirine in Patients with HIV, Hepatitis B Core Antibody Positivity, and Hepatitis B Surface Antigen Negativity

**DOI:** 10.1093/ofid/ofac492.106

**Published:** 2022-12-15

**Authors:** Elliott Welford, Jeffrey Yin, Lucas Hill, Darcy Wooten

**Affiliations:** University of California, San Francisco, San Francisco, CA; University of California, San Diego, San Diego, California; University of California, San Diego, San Diego, California; University of California, San Diego, San Diego, California

## Abstract

**Background:**

Two-drug antiretroviral therapy (ART), including long-acting injectable cabotegravir and rilpivirine (LAI CAB/RPV), is increasingly used for treatment of people with HIV. Many two-drug regimens do not contain tenofovir (TFV), and guidelines recommend ensuring patients are not chronically infected with hepatitis B virus (HBV) before switching to a TFV-free regimen since this could lead to HBV reactivation. The approach to regimen switching in patients who are HBV surface antigen (sAg) negative but who are HBV core antibody (HBcAb) positive is less clear. We describe three cases of HBV low-level viremia in patients switched to LAI CAB/RPV and who were HBcAb positive and sAg negative.

**Methods:**

We conducted a retrospective case series of patients with HIV switching to LAI CAB/RPV who were HBcAb positive and sAg negative. We reviewed pre-switch HBV studies, history of HBV vaccination, current ART, CD4 cell count, and HIV viral load, and liver function tests (LFTs) where available. After switching to LAI CAB/RPV, we reviewed patients’ HBV DNA PCR, HBVsAg, and ART management.

**Results:**

Of the 149 patients in our clinic who were switched to LAI CAB/RPV, 38 (25.5%) were HBcAb positive and sAg negative. Of these 38 patients, three (7.9%) developed HBV viremia (Figure 1). Two of the patients were switched back to a TFV-containing three-drug regimen with subsequent suppression of HBV DNA levels. Through shared decision-making, the third patient opted to continue LAI CAB/RBV. Repeat HBV DNA levels were detected but remained low and the patient’s sAg remained negative. All patients’ LFTs remained within normal limits. Of interest, one of the three patients had a surface antibody level of 115 mIU/mL at baseline.

**Conclusion:**

Our case series describes three cases of HBV low-level viremia following a switch off of HBV-active ART to LAI CAB/RPV. The clinical significance of this low-level viremia is unclear and warrants further investigation. We recommend a post-hoc analysis of stored samples from patients in ATLAS, FLAIR, and ATLAS-2M who were HBcAb positive to determine if low-level HBV viremia occurred in these patients and if surface antibody levels influenced the likelihood of reactivation.

**Disclosures:**

**Lucas Hill, PharmD, AAHIVP**, Gilead Sciences: Speakers Bureau.

**Figure 1.**
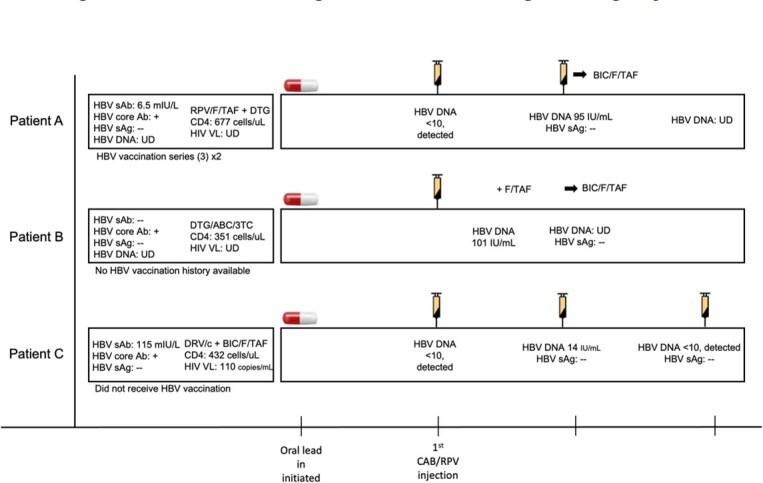
Baseline patient characteristics and timeline trend of hepatitis B DNA and hepatitis B surface antigen levels following a switch to long-acting injectable cabotegravir/rilpivirine.

